# Supplementary material for: Survival prediction in gliomas based on MRI radiomics combined with clinical factors and molecular biomarkers
Source: PeerJ. 2025 Aug 20;13:e19906. doi: 10.7717/peerj.19906 (PMC12374691; doi:10.7717/peerj.19906)
Supplement: Supplemental Information 2 [file peerj-13-19906-s002.doc]

Original code annotation submitted for the first time (starting from "setwd('F:/')" and ending at "summary (time_roc_res)")

The line number is automatically generated after R software opens the code file.

Line 70 #xvar为x轴命名 #"xvar" denotes a variable named for the X-axis

Line 115 #调整字体大小 #Adjust the font size

Line 133 #设置调色板 #Set the color palette

Line 201 #图例位置 #Legend location

#Legend location: This note should be placed after legend =' top'. I sincerely apologize for the previous oversight where it was positioned after legend.title=' '.

Line 209 #统计学方法文本的位置 #Location of statistical methods text

Line 210 #设置调色板 #Set the color palette

Line 247 #error bar的粗细 #The thickness of the error bar

Line 248 #error bar的类型，可以是0-6 #The type of error bar can be 0-6

Line249 #error bar的颜色 #The color of the error bar

Line 253 #字的大小 #The size of the font

Line 255 #连线的类型，可以是"p","b","o" #The type of connection can be "p","b","o"

Line 256 #连线的粗细 #The thickness of the connection lines

Line 257 #点的形状，可以是0-20 #The shape of the point can range from 0 to 20

Line 258 #连线的颜色 #The color of the connection line

Line 260 #边框粗细 #Border thickness

Line 261 #对角线为虚线 #The diagonal is a dotted line

Line 262 #对角线的粗细 # The thickness of the diagonal

Line 263 #对角线的颜色 #The color of the diagonal

Line 272 #error bar的粗细 #The thickness of the error bar

Line 273#error bar的类型，可以是0-6 #The type of error bar can be 0-6

Line 274 #error bar的颜色 #The color of the error bar

Line 278 #字的大小 #The size of the font

Line 280 #连线的类型，可以是"p","b","o" #The type of connection can be "p","b","o"

Line 281 #连线的粗细 #The thickness of the connection lines

Line 282 #点的形状，可以是0-20 #The shape of the point can range from 0 to 20

Line 283 #连线的颜色 #The color of the connection line

Line 285 #边框粗细 #Border thickness

Line 286 #对角线为虚线 #The diagonal is a dotted line

Line 287 #对角线的粗细 # The thickness of the diagonal

Line 288 #对角线的颜色 #The color of the diagonal

Line 297 #error bar的粗细 #The thickness of the error bar

Line 298 #error bar的类型，可以是0-6 #The type of error bar can be 0-6

Line 299 # error bar的颜色 #The color of the error bar

Line 303 #字的大小 #The size of the font

Line 305 #连线的类型，可以是"p","b","o" #The type of connection can be "p","b","o"

Line 306 #连线的粗细 #The thickness of the connection lines

Line 307 #点的形状，可以是0-20 #The shape of the point can range from 0 to 20

Line 308 #连线的颜色 #The color of the connection line

Line 310 #边框粗细 #Border thickness

Line 311 #对角线为虚线 #The diagonal is a dotted line

Line 312 #对角线的粗细 #The thickness of the diagonal

Line 313 #对角线的颜色 #The color of the diagonal

Code annotation submitted for the second time after major revision (starting from "#Set working directory" and ending at "dev.off ()")

Line 447 #xvar为x轴命名 #"xvar" denotes a variable named for the X-axis

Line 604 #图例位置 #Legend location

#Legend location: This note should be placed after legend =' top'. I sincerely apologize for the previous oversight where it was positioned after legend.title=' '.

Line 612 #统计学方法文本的位置 #Location of statistical methods text

Line 613 #设置调色板 #Set the color palette

Line 837 #调整字体大小 #Adjust the font size

Line 840 #图例位置 #Legend location

#Legend location: This note should be placed after legend =' top'. I sincerely apologize for the previous oversight where it was positioned after legend.title=' '.

Line 846 #调整P值位置 #Adjust the p-value position

Line 848 #设置调色板 #Set the color palette
