# Supplementary material for: Survival prediction in gliomas based on MRI radiomics combined with clinical factors and molecular biomarkers
Source: PeerJ. 2025 Aug 20;13:e19906. doi: 10.7717/peerj.19906 (PMC12374691; doi:10.7717/peerj.19906)
Supplement: Supplemental Information 7 [file peerj-13-19906-s007.pdf]

The constructed radiomics signature

The radiomics signature constructed by linear fitting based on the coefficient weights of the 31 radiomics features was as follows:

Risk score = CE-T1WI_exponential_firstorder_10Percentile×0.09646＋CE-T1WI_exponential_glcm_Correlation×(-0.55328)+CE-T1WI_exponential_glcm_MCC×0.31417＋CE-T1WI_lbp.3D.m1_firstorder_Median×(-0.15658)＋CE-T1WI_lbp.3D.m1_glcm_ClusterShade×0.2069＋CE-T1WI_lbp.3D.m2_glrlm_RunEntropy×0.15347＋CE-T1WI_lbp.3D.m2_glszm_ZoneEntropy×0.16898＋CE-T1WI_logarithm_firstorder_Kurtosis×(-0.09303)＋CE-T1WI_original_glcm_ClusterShade×(-0.10796)＋CE-T1WI_original_glszm_LowGrayLevelZoneEmphasis×(-0.03859)＋CE-T1WI_square_glrlm_RunLengthNonUniformity×0.06094＋CE-T1WI_wavelet.LLH_glcm_JointEnergy×(-0.02118)＋CE-T1WI_wavelet.LHH_gldm_DependenceEntropy×0.11185＋CE-T1WI_wavelet.HHL_firstorder_Kurtosis×(-0.00051)＋CE-T1WI_wavelet.HHL_glcm_MCC×(-0.17998)＋CE-T1WI_wavelet.HHL_glrlm_ShortRunHighGrayLevelEmphasis×0.16793＋CE-T1WI_wavelet.HHH_glcm_MCC×(-0.10809)＋CE-T1WI_wavelet.LLL_glcm_JointAverage×0.14795＋CE-T1WI_wavelet.LLL_glrlm_ShortRunLowGrayLevelEmphasis×(-0.01716)＋CE-T1WI_wavelet.LLL_glszm_LowGrayLevelZoneEmphasis× (-0.15336)＋T2FLAIR_exponential_glcm_ClusterTendency×0.00633＋T2FLAIR_gradient_glcm_DifferenceVariance×(-0.15272)＋T2FLAIR_gradient_glszm_SmallAreaLowGrayLevelEmphasis×0.00004＋T2FLAIR_lbp.2D_glcm_Autocorrelation×(-0.07774)＋T2FLAIR_lbp.3D.m2_glcm_Autocorrelation×(-0.05196)＋T2FLAIR_lbp.3D.m2_glcm_ClusterShade×0.15457＋T2FLAIR_lbp.3D.k_firstorder_Variance×(-0.03765)＋T2FLAIR_lbp.3D.k_gldm_LargeDependenceHighGrayLevelEmphasis×0.05898＋T2FLAIR_wavelet.LLH_glcm_Imc2×(-0.22901)＋T2FLAIR_wavelet.LHH_glcm_Imc2×(-0.01516)＋T2FLAIR_wavelet.LLL_glszm_HighGrayLevelZoneEmphasis×(-0.19333).

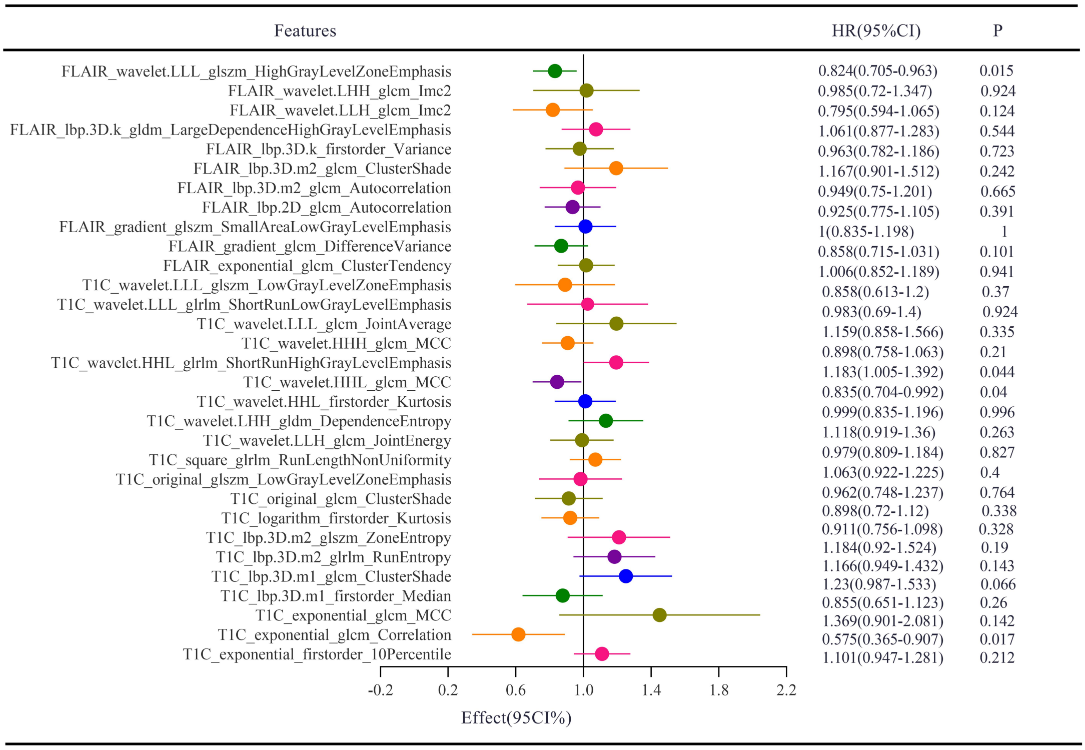

Figure S1. HR values with 95% CI and p values of each selected feature of the radiomics model on the forest plot in the training cohort
